# The mechanoelectrical transducer channel is not required for regulation of cochlear blood flow during loud sound exposure in mice

**DOI:** 10.1038/s41598-020-66192-6

**Published:** 2020-06-08

**Authors:** George W. S. Burwood, Suzan Dziennis, Teresa Wilson, Sarah Foster, Yuan Zhang, Gangjun Liu, Jianlong Yang, Sean Elkins, Alfred L. Nuttall

**Affiliations:** 1 Oregon Hearing Research Center, Dept. of Otolaryngology / HNS, Oregon Health & Science University, 3250S.W. Sam Jackson Park Rd., Portland, OR 97239 USA; 2 Shenzhen Bay laboratory, 5F, No.9 Duxue Rd., Nanshan District, Shenzhen, Guangdong, China; 3 Ningbo Institute of Materials Technology and Engineering, No. 1219 Zhongguan West Road Zhenhai District, Ningbo City, Zhejiang Province 315201 P.R. China

**Keywords:** Auditory system, Circulation

## Abstract

The mammalian cochlea possesses unique acoustic sensitivity due to a mechanoelectrical ‘amplifier’, which requires the metabolic support of the cochlear lateral wall. Loud sound exposure sufficient to induce permanent hearing damage causes cochlear blood flow reduction, which may contribute to hearing loss. However, sensory epithelium involvement in the cochlear blood flow regulation pathway is not fully described. We hypothesize that genetic manipulation of the mechanoelectrical transducer complex will abolish sound induced cochlear blood flow regulation. We used *salsa* mice, a *Chd23* mutant with no mechanoelectrical transduction, and deafness before p56. Using optical coherence tomography angiography, we measured the cochlear blood flow of *salsa* and wild-type mice in response to loud sound (120 dB SPL, 30 minutes low-pass filtered noise). An expected sound induced decrease in cochlear blood flow occurred in CBA/CaJ mice, but surprisingly the same sound protocol induced cochlear blood flow increases in *salsa* mice. Blood flow did not change in the contralateral ear. Disruption of the sympathetic nervous system partially abolished the observed wild-type blood flow decrease but not the *salsa* increase. Therefore sympathetic activation contributes to sound induced reduction of cochlear blood flow. Additionally a local, non-sensory pathway, potentially therapeutically targetable, must exist for cochlear blood flow regulation.

## Introduction

Sensitive hearing provided by normal cochlear function is energy demanding and requires constant and tightly regulated delivery of oxygen, glucose and other nutrients^1–4^. These metabolites can reach the cochlear fluids which bathe the organ of Corti, via the radiating arterioles and capillary beds of the cochlear lateral wall. There, the capillaries distribute to two systems, one serving the spiral ligament while the other is to the stria vascularis. The stria vascularis has both a blood-labyrinth barrier and possesses an electrogenic process which maintains the endocochlear potential (EP).

Cochlear blood flow is responsive to sound stimulation^5^. Intravital microscopy studies show that lateral wall capillaries dilate, facilitated by lateral wall pericyte signaling, during physiologically relevant sound exposure^6^. In contrast, cochlear blood flow declines in response to damaging sound levels (>100 dB SPL), which quickly cause noise induced hearing loss^5,7,8^. Sympathetic neuronal innervation regulates cochlear blood flow in the main arterioles upstream of the lateral wall. This sympathetic innervation is supplied by the superior cervical ganglion (SCG) and the stellate ganglion. Electrical stimulation of the SCG^9^ and the stellate ganglion^10,11^ results in cochlear blood flow decline and pharmacological blockade of adrenergic receptors abolishes the effect.

The above investigations suggest that two regulatory systems influence cochlear blood flow during sound exposure. 1) A pericyte mediated lateral wall feedback loop linking sound induced metabolic demand to vascular parameters, i.e. flow, and 2) a global, sympathetically activated arteriolar feedback loop. To the best of our knowledge, no one has directly investigated the activation and balance of the two systems, and their relationship to organ of Corti activation. We therefore assume that due to their tight coupling to acoustic stimulation, both systems rely upon activation of the sensory hair cells which contain sound activated mechanoelectrical transducer (MET) channels. Hence, it is the aim of this study to reveal whether the MET channel is required for cochlear blood flow regulation.

**Figure 1:**
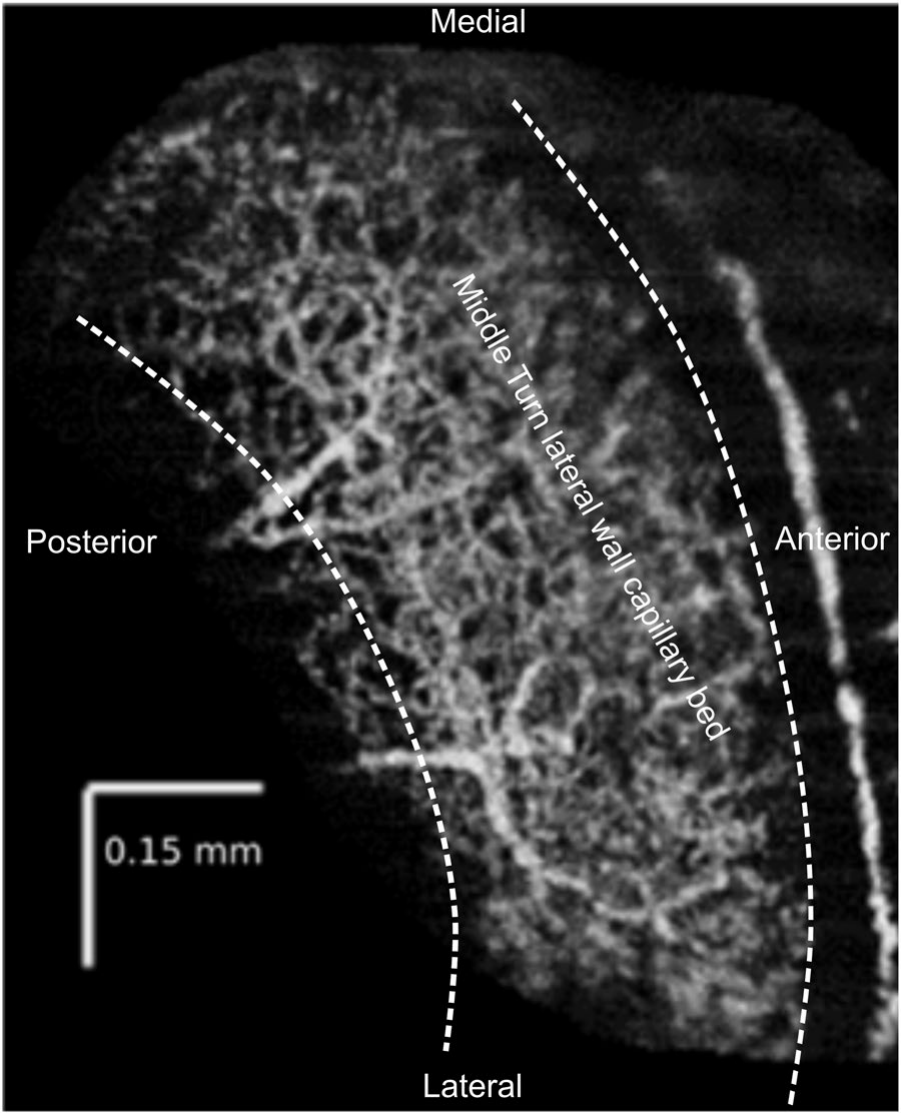
En face reconstruction of the ventral view of lateral wall blood flow in the middle turn of a murine left cochlea using OCTA. The middle turn organ of Corti (not visible) runs mediolaterally from low to high frequency along the cochlear spiral, between the two dashed lines, and includes the approximate location of a typical region of interest selection for flow analysis. This image is an average of 5 consecutive scans in the absence of sound.

To establish the effect of MET channel function on cochlear blood flow, we used *salsa* mice. *Salsa* mice express a point mutation in a Ca^2+^-binding motif of cadherin23 (Cdh23), a vital component of normal tip links, which facilitate activation of the MET channel when sound stimulates the hair cells^12^. These mice show progressive tip-link loss, and eventual organ of Corti degeneration, rendering these animals profoundly deaf. Loss of tip links naturally results in the loss of sound evoked MET channel activity, silencing both inner hair cells (IHCs) and outer hair cells (OHCs). We reasoned that if the MET channel is required for regulation of cochlear blood flow, the lateral wall blood flow of *salsa* mice will not change in response to loud sound exposure, in comparison to control. In control animals, we expect the previously reported loud sound exposure induced progressive decrease in cochlear blood flow^5^. We used spectral domain optical coherence tomography angiography (OCTA)^5,13,14^ (Fig. 1) to measure relative changes in cochlear blood flow at the capillary level. We present evidence to suggest that the MET channel is required for the interaction between sympathetic neural mechanisms and the cochlea, but not for local control of cochlear blood flow.

## Results

### *Salsa* mice on the CBA/J background do not experience hair cell loss in the middle turn of the cochlea, but are profoundly deaf at p56

**Figure 2:**
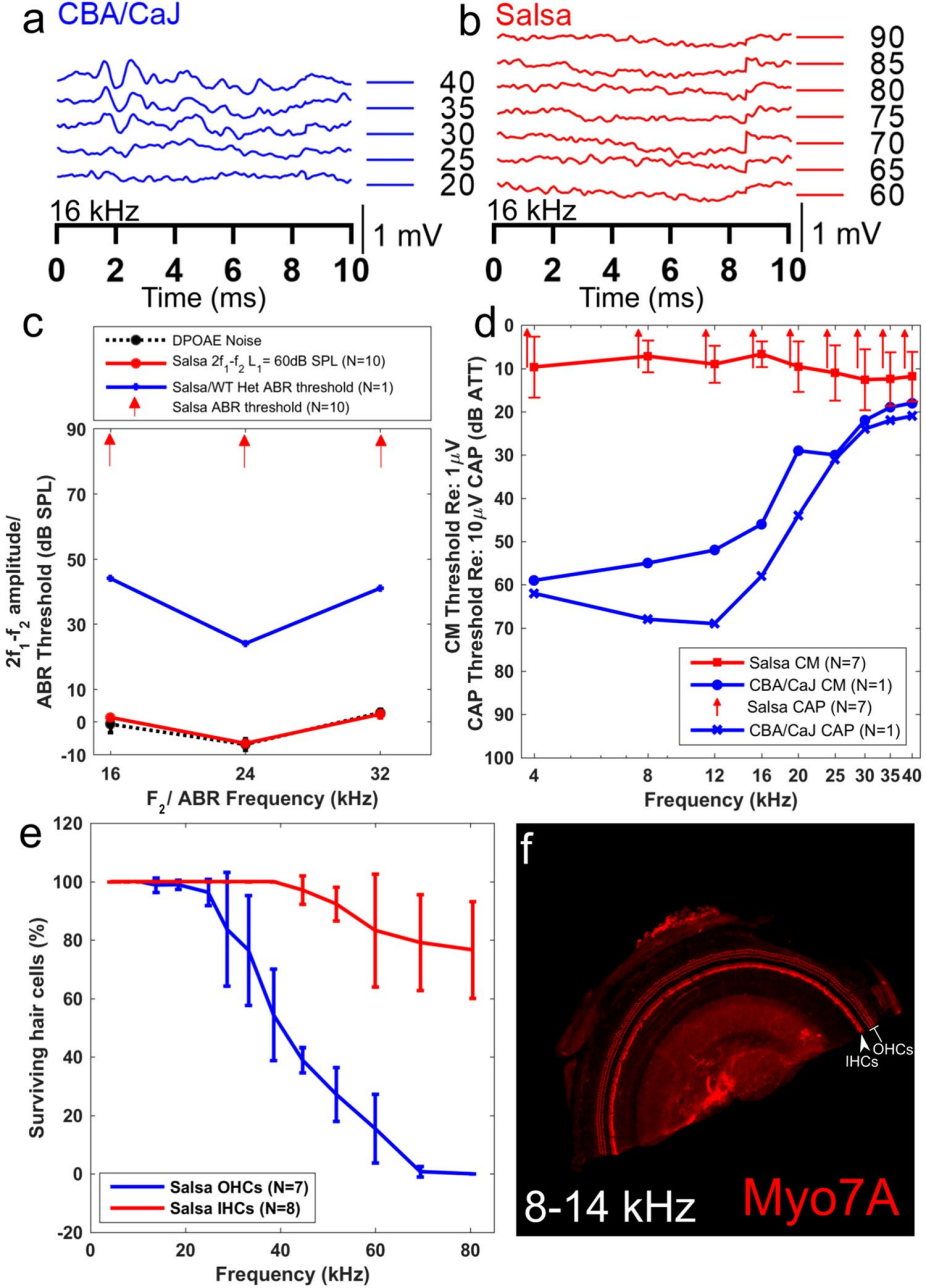
Characterization of hearing function in the *salsa* mouse. (**a**) Example ABR time domain response to a 16 kHz tone, for an 8 week old CBA/CaJ mouse. The threshold was identified to be 30 dB SPL. (**b**) Example ABR time domain response to a 16 kHz tone for an 8 week old *salsa* mouse. There was no identifiable response at the highest level tested (90 dB SPL). (**c**) 2f1-f2 amplitudes (L1 = L2 + 10 dB=60 dB SPL, red circles solid line), ABR thresholds for 16, 24 and 32 kHz, for an example heterozygous *salsa* mouse (blue plusses solid line) and homozygous *salsa* mice (N = 10, red arrows). There was no ABR response and no 2f1-f2 signal for the *salsa* mice. (**d**) CAP and CM measurements for an example CBA/CaJ mouse (CAP: blue crosses, CM: blue circles) and *salsa* mice (N = 7). There was no measurable CAP at any attenuation value for the *salsa* mice (red arrows). No CM signal specific to the cochlea was measured (red squares solid line). (**e**) IHC (N = 8, red line) and OHC (N = 7, blue line) survival for 8 week old salsa mice. 98.8 ± 2.5%, 98.9 ± 1.6% and 96.4 ± 4.5% of the OHCs and 100% of the IHCs survived at the 14, 18 and 25 kHz locations respectively. (**f**) An example of an 8–14 kHz organ of Corti wholemount from an 8 week old *salsa* mouse, stained with Myosin 7 A (red). The OHCs and IHCs are largely intact in this location (labelled).

 It has previously been observed that *salsa* mice are profoundly deaf by p60^12^. To confirm that MET channel activity was abolished in our backcrossed *salsa* mice at 7–8 weeks of age, we screened their hearing with ABR and 2f1-f2 DPOAEs. A cohort of 28 mice underwent screening at various ages above p30. The screening measurements for a subset, closest to the age at which they were used for blood flow experiments is shown in Figure 2. Figure 2a shows a typical ABR waveform to a 16 kHz tone, for a CBA/CaJ mouse. A clear wave I response can be seen at 30 dB SPL. Figure 2b shows the same measurement from a *salsa* animal. There is no wave I response even at 90 dB SPL. ABR thresholds (N = 10, red arrows Fig. 2c) and 2f1-f2 distortion (N = 10, red circles solid line Fig. 2c) were abolished in *salsa* mice at 7–8 weeks of age, implying profound hearing loss. An example wild-type mouse showed a normal range of hearing (blue plusses solid line, Fig. 2c).

To confirm the above findings, the compound action potential (CAP, the field potential produced by the summated response of the auditory nerve) and cochlear microphonic (CM, the field potential produced by summated OHC mechanoelectrical transduction) were measured on the round window membrane of *salsa* mice. An example wild-type mouse is included for comparison. In *salsa* mice, CAP was absent (N = 7, red arrows Fig. 2d), and the cochlear microphonic (CM) could not be evoked at any threshold tested by stimuli up to the linear limit of the sound system (N = 7, red squares, solid line Fig. 2d). Similar CM thresholds were measured when the electrode was placed upon muscle away from the cochlea, indicating a non-specific response at high sound levels. In contrast, an example control which was heterozygous for the *salsa* mutation showed robust CAP and CM thresholds (blue crosses and circles respectively, solid lines Fig. 2d).

The endocochlear potential for *salsa* mice at 7–8 weeks of age was normal: 112 +/− 4 mV (N = 4). Thus, the age at which we investigated loud sound exposure influence on cochlear blood flow in these animals was optimal to test an organ of Corti in the absence of MET channel induced metabolism and auditory nerve activity, but which was otherwise sufficiently intact.

*Salsa* mice gradually lose their IHC and OHC tip links with age, beginning with the basal hair cells, and by p60 the vast majority of the tip links have degenerated^12^. However, the mouse experiences early (p30) basal OHC loss and this begins before tip link loss is complete. For our interpretation of the effects of tip-link loss to be accurate and not confounded by OHC loss^15^, we counted HC populations in *salsa* mice at or near p56 (Fig. 2e,f). The data show that approximately 98% of the OHCs were present (N = 7) in the region of interest for cochlear blood flow studies (blue line, Fig. 2e). In addition, a majority of OHCs survived, approaching the lower basal turn of the cochlear partition (approximately 40 kHz CF). IHCs were mostly intact (N = 8) up to the highest frequencies examined (approximately 80 kHz). This phenotype ensures that MET channel activity loss could be the major contributor to the change in cochlear blood flow phenotype, rather than unknown confounding effects due to the loss of the sensory epithelium altogether. Additionally, lateral wall blood flow shows tonotopic sensitivity to loud sound exposure^7^, and so the survival of the organ of Corti adjacent to the OCTA measurement site is assumed to influence the lateral wall locally.

### Loud sound exposure induced changes in cochlear blood flow in the 12–16 kHz tonotopic place associated lateral wall of wild-type and *salsa* mice

**Figure 3:**
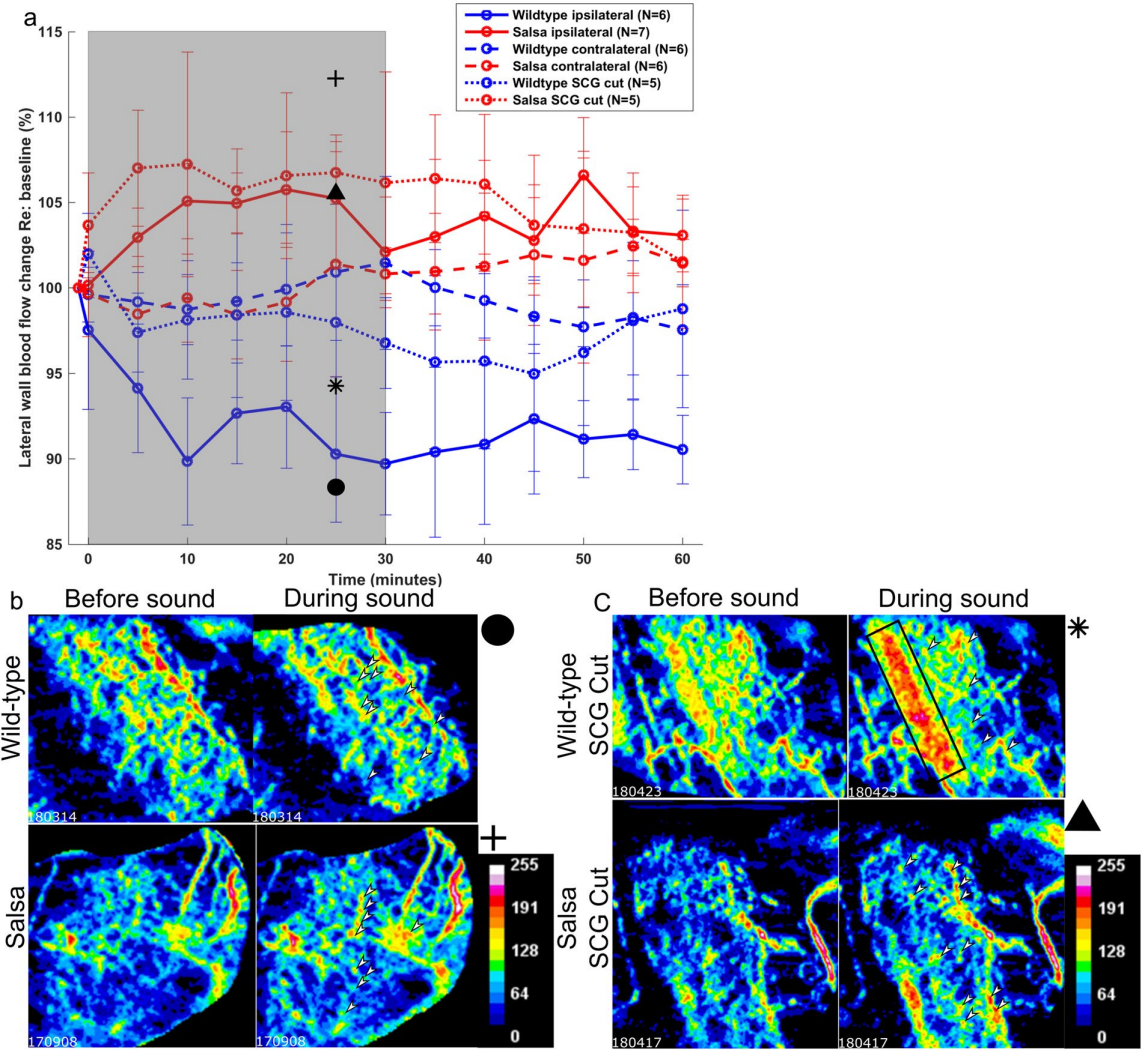
(**a**) Effect of genotype, stimulated ear, and sympathectomy on cochlear blood flow. Relative cochlear blood flow change during and after ipsilateral loud sound exposure in wild-type (N = 6, blue circles, solid line) and *salsa* (N = 7, red circles, solid line) mice, and during and after contralateral loud sound exposure in wild-type (N = 6, blue circles, dashed line) and *salsa* (N = 6, red circles, dashed line) mice. MET channel function was a statistically significant factor in cochlear blood flow change between the ipsilateral groups (rmANOVA, F = 7.448, p = <0.01). Neither MET channel function (rmANOVA, F = 1.076, p = 0.381) nor time (rmANOVA, F = 1.148, p = 0.348) significantly affected contralateral cochlear blood flow. Wild-type mice with the anterior branches of the SCG transected (N = 5, blue circles, dotted line) show significantly reduced cochlear blood flow decline during loud sound exposure compared to intact wild-type mice (rmANOVA, F = 3.043, p = 0.02). There was no statistically significant effect on cutting anterior branches of the SCG of *salsa* mice (N = 5 red circles, dotted line) compared to intact *salsa* mice (rmANOVA, F = 0.841, p = 0.510). Loud sound exposure is indicated using the shaded area of the graph. (**b**) Examples, selected at random, of OCTA scans for wild-type (upper panels) and *salsa* mice (lower panels) before (left) and after 25 minutes (right) of sound exposure. (**c**) as in Fig. 3b but for wild-type (upper panels) and *salsa* (lower panels) after sectioning of the anterior branches of the SCG. A sound induced flow artifact representing organ of Corti vibration (black rectangle, upper left panel) is shown, and was excluded from the ROI. For Fig. 3b,c, black symbols denote the position of the individual blood flow value on the graph in Fig. 3a. The color bar denotes arbitrary 8 bit pixel intensity in false color. For all during sound images (3 b, c, right hand panels), white arrows indicate example areas of blood flow change relative to before sound (3 b, c, left hand panels).

To determine the effects of the loss of MET channel activity of cochlear blood flow regulation, we subjected *salsa* and wild-type age matched controls to an acute loud sound exposure protocol, as described in the methods. As anticipated, the mean cochlear blood flow of wild-type mice (N = 6) decreased during the exposure time (Fig. 3a, blue circles, solid line, exposure time is shaded in gray). The decrease plateaued after approximately 10 minutes of loud sound exposure, at 89.8 ± 3.7%. Cochlear blood flow did not recover to baseline during the 30 minute post-loud sound exposure period.

Surprisingly, the blood flow in the lateral wall of the *salsa* animals (N = 7) increased in response to sound (Fig. 3a, red circles, solid line), decreasing towards, but not reaching baseline after loud sound exposure offset. MET channel function had a statistically significant effect upon cochlear blood flow (rmANOVA, F = 7.448, p = «0.01). The magnitude of the increase was smaller than that of the decrease observed with the wild-type group (5.75 ± 3.4% vs −10.2 ± 3.7%). The increase in cochlear blood flow observed with the *salsa* group appeared to be more closely related to the loud sound exposure duration than the decrease of the wild-type group. This experiment indicates that loss of MET channel function does not abolish cochlear blood flow regulation in response to loud sound exposure, but is necessary for the wild-type cochlear blood flow reduction phenotype. This does not support our initial hypothesis that the MET current is the drive linking sound evoked organ of Corti related vasoactivity in the lateral wall.

Figure 3b shows randomly selected examples of OCTA scans before and during (at 25 minutes) sound exposure, from wild-type (upper panels) and *salsa* (lower panels) animals. Symbols, to the right of these panels (wild-type: black circle, *salsa*: plus sign) show where the individual blood flow values fall in the population data of Fig. 3a.

### Contralateral loud sound exposure did not induce measurable changes in cochlear blood flow in either group

In *salsa* mice, sound exposure does not induce any HC MET current mediated afferent signal to the brain, although contributions from the vestibular system and non-MET conductances are likely intact. However, the above experiments indicate that a loss of MET driven auditory nerve stimulation may alter cochlear blood flow regulation. We therefore sought to identify whether systemic norepinephrine and/or epinephrine could be responsible for the wild-type phenotype. To do this, we repeated our experiments as above, the only modification being that the loud sound exposure would be supplied to the contralateral ear,  while we measured the ipsilateral ear for cochlear blood flow changes (Fig. 3a, dashed lines). Systemic effects of loud sound exposure, for example blood pressure change, should influence cochlear blood flow in the non-stimulated ear. There was no consistent change in cochlear blood flow during or after loud sound exposure in the contralateral ear of either group. Genotype had no statistically significant effect upon cochlear blood flow during contralateral stimulation (rmANOVA, F = 1.076, p = 0.381). Time was also not a statistically significant effect (rmANOVA, F = 1.148, p = 0.348), indicating that the values did not depart significantly from baseline. These results suggest that the wild-type phenotype is not induced by a circulated ligand.

### Unilateral sympathectomy partially abolishes cochlear blood flow reduction in wild-type mice, but does not alter the *salsa* cochlear blood flow phenotype

Electrical stimulation of the SCG reduces cochlear blood flow^9^. There are no studies to date which report cochlear blood flow during loud sound exposure in the absence of the SCG with the exception of^8^ which utilizes part of the dataset reported here. We reasoned that adrenergic activity in the cochlea during loud sound exposure may induce vasoconstriction, thus slowing cochlear blood flow, and that this sympathetic activity is responsible for the wild-type cochlear blood flow phenotype.

A separate cohort of *salsa* and wild-type mice underwent acute unilateral sympathectomy by removal of the ipsilateral SCG during the surgery to expose the cochlea. They were then noise exposed as previously. The data (Fig. 3a, red and blue circles respectively, dotted lines) show that sympathectomy significantly reduced the loud sound exposure induced cochlear blood flow decline seen in wild-type, but only for data points acquired during loud sound exposure (rmANOVA, F = 3.043, p = 0.02). This indicates that the sympathectomy delayed and/or reduced the putative sympathetic effect, but did not abolish it entirely.

There was no statistical difference between the loud sound exposure induced cochlear blood flow increase of desympathetized *salsa* mice and their intact controls (rmANOVA, F = 0.841, p = 0.510). Since in *salsa* mice there is no MET channel mediated auditory nerve activity, and thus the SCG is likely not stimulated, this is the expected result. The sympathetic nervous system therefore influences cochlear blood flow, but only in the presence of a functional MET channel.

Figure 3c shows randomly selected examples of OCTA scans before and during (at 25 minutes) sound exposure, from wild-type (upper panels) and *salsa* (lower panels) animals after sympathectomy. The upper panel during sound exposure of Fig. 3c shows an example of sound induced artifact which was excluded from the region of interest (ROI) for this animal, and all other animals where an instantaneous, sound dependent, localized increase in flux signal was observed close to the expected location of the organ of Corti or Reissner’s membrane. The associated symbols to the right of the panels (wild-type SCG cut: black asterisk, *salsa* SCG cut: black triangle) show the data in context in Fig. 3a.

## Discussion

### Summary of findings

This study has  four main conclusions. We confirm that CBA/CaJ (wild-type) mice exposed to ipsilateral loud sound exposure show the commonly found ipsilateral reduction in cochlear blood flow. Loud sound exposure contralateral to the measured ear did not change cochlear blood flow in wild-type or *salsa* mice, possibly indicating negligible crosstalk via bone conduction with the loud sound exposure utilized, as well as an absence of influence of sound on systemic circulation. The surprising principal result of our work is that regulation of cochlear blood flow in response to loud sound exposure does not depend upon hair cell transduction current flow, and the consequent metabolic ‘load’ from the activation of the sensory epithelium. Additionally, manipulation of the adrenergic inputs to the cochlea confirm that the sympathetic nervous system dominates cochlear blood flow regulation in response to intense loud sound exposure in the wild-type mouse.

### Normal MET channel function is not necessary for cochlear blood flow regulation, but MET channel dysfunction changes the nature of that regulation

Moderate level loud sound exposure has been shown to increase cochlear blood flow. Laser Doppler flowmetry (LDF) measurements show increases in cochlear blood flow at 85 dB SPL, becoming decreases at 125 dB SPL, when measured from the basal turn^16^. Intravital microscopy in guinea pigs with normal hearing shows that cochlear blood flow increases in response to a pure tone of 500 Hz at 85 dB SPL delivered for 10 minutes^6^. The observation of higher red blood cell velocity is accompanied by Ca^2+^ aggregation in pericytes, which are a component of the fibrovascular unit of the lateral wall and spiral ligament^17^. It is likely that this effect is mediated by vasodilatory agents such as nitric oxide^18,19^. Similar Intravital microscopy approaches also show that cochlear blood flow increases occur in guinea pig lateral walls, resulting from 30 minutes of noise at 85 dB SPL^20^, and for a higher noise level (110 dB SPL). This increase was followed by a sharp decrease during loud sound exposure. In general, this is at odds with multiple observations, which report decreases in cochlear blood flow^21^. It is possible that interpretation of the 110 dB SPL result is complicated by the choice of pentobarbital anesthesia, which interacts with the adrenergic system^22^.

Here, we provide the novel observation that a local mechanism may possibly increase cochlear blood flow in the *salsa* mouse in response to loud sound exposure, independent of the MET current. This is not likely an effect of loss of amplification, which would be entirely saturated in both control and *salsa* at 120 dB SPL. The observed independence of blood flow regulation from MET current has wide ranging significance due to the importance of the MET channel in HC hair cell physiology. It is supposed that the downstream effects of loud sound exposure including lateral wall vascular and fibrocyte pathology^23^, the dynamic ionic environment of the scala media^24^, and indeed metabolic regulation^25^ are dependent upon normal mechanoelectrical transduction as a step in a feedback loop between the organ of Corti and lateral wall. Non-MET, pathways of ion flux from endolymph though the sensory epithelium have not been observed^26^ unless the reticular lamina is breached by acoustic overstimulation^23^. The MET channel therefore represents the only sound coupled, inwardly rectifying conductance of ions between scala media and scala tympani, thereby facilitating ion recycling through the lateral wall via supporting cells, under both normal and pathological sound conditions. Any point along this pathway could contribute to the effects upon cochlear blood flow regulation reported here. If metabolic stress does not contribute to cochlear blood flow regulation, it is possible that direct mechanical stress may constitute a regulatory pathway, either at the organ of Corti, or in the lateral wall. Stretch sensitive ion channels^27–29^ may play a role in this response, especially given the intensity of the stimulus used in this study.

It is important to note that we are aware of no evidence of *in vivo* mechanical gating of MET channels without tip links. Recently, *in vitro* liposomal analysis of a truncated form of TMC1, a putative MET channel pore protein, showed some intrinsic mechanosensitive properties^30^. However, this activity may be due to the truncation of the N-terminal domain, necessary to permit efficient TMC1 migration to the liposomal membrane. Tip link lower protein PCDH15 interaction with TMC1 is dependent upon this domain^31^. As such, the mechanosensitive properties of truncated TMC1 are not likely to represent the activity of native MET channels, as the N-terminal domain may contribute to maintenance of channel closure. The blood flow response observed in *salsa* mice is therefore unlikely to be related to non-physiological MET function.

### Sympathetic activity may underlie the wild-type cochlear blood flow decrease during loud sound exposure

The decrease in cochlear blood flow response to loud sound exposure recorded here in wild-type mice is consistent with recordings made in other species. Loud sound exposure decreases cochlear blood flow when measured by LDF in guinea pigs^7^ and Doppler optical microangiography in mice^5^. What mechanism, then, is responsible for this phenotype? It is likely that the sympathetic nervous system plays a role.

There is a hypothetical neuronal interface between the afferent auditory pathway and the sympathetic nervous system^32^, and it is known that cochlear blood flow is in part regulated by sympathetic activity^33,34^. Some of the neuronal outputs from the SCG follow the internal auditory artery, and associate with cochlear blood vessels^35^, although this is controversial. α 1 adrenergic receptors^36^, norepinephrine^37^, and tyrosine hydroxylase, the rate limiting enzyme for norepinephrine synthesis^38^, are also present in cochlear blood vessels. Additionally, SCG activation by electrical stimulation reduces cochlear blood flow^33^, which is the expected result of α 1 receptor activation. Our wild-type loud sound exposure experiments with sympathectomy suggest adrenergic activation is a contributing factor to cochlear blood flow reduction. Latent reduction still present after our partial sympathectomy may be due to stellate ganglion effects. Other, non-neuronal agents such as Tumor Necrosis Factor α^39^, ROS or 8-isoprostane^40^ are known to locally induce vasoconstriction, including in response to loud sound exposure^41,42^ and so these likely contribute to the reduction phenotype. Whether these pathways would be activated in *salsa* mice is unknown, but the strong vasodilation phenotype observed implies that they are not.

With this in mind, the current *salsa* mouse model should represent total absence of acoustic activation of both stellate ganglion and SCG, i.e. the model is functionally equivalent to total sympathectomy. As a result, cutting the SCG had no overall effect upon cochlear blood flow in *salsa* mice. In support of^33,34^, the resulting absence of loud sound exposure induced cochlear blood flow reduction leads to the conclusion that intense loud sound exposure induces MET channel mediated activation of adrenergic fibers in the cochlea.

### Contralateral stimulation indicates that loud sound exposure induced cochlear blood flow changes are effects local to the cochlea

The variations in cochlear blood flow of the ear contralateral to loud sound exposure were insignificant compared to the non-loud sound exposure controls^5^, confined to a few % either side of base line. Firstly, this suggests that bone conduction of the contralateral stimulus was insufficient to cause a statistically significant shift from baseline cochlear blood flow. Secondly, these results are in agreement with the findings that loud sound exposure does not induce blood pressure changes in anaesthetized guinea pigs^7^ and rats^43^ that would overcome local cochlear blood flow homeostasis mechanisms^44^, especially under ketamine based anesthesia^45^. Therefore, the cochlear blood flow changes measured in the ipsilateral stimulation experiments reported here reflect ipsilateral cochlear arteriolar and lateral wall vascular status, not circulated ligand effects.

### Evolutionary implications of sympathetically induced reduction of cochlear blood flow during loud sound exposure

The broader implications of the results described in this work are further highlighted when examined through the evolutionary lens. Sympathetic control of cochlear blood flow is clearly present, although its role is unclear. Regardless of the purpose of this system, both stress and loud sound exposure can affect hearing and intense loud sound exposure may induce the same sympathetic effects on the cochlea as physiological stress^46^. For example, cochlear catecholamine concentration and neural hearing thresholds increase in response to emotional stress^47^ and administration of catecholamines to the cochlea induces neural hearing threshold increase^48^.

While physiological stress was doubtless induced by many adaptation pressures present during the evolution of the cochlea, sustained loud sound exposure akin to that which society experiences today was absent. We speculate that the resulting demand imposed upon cochlear metabolism by moderate sound is met by the pericyte mediated system that has been unmasked in *salsa* mice. Conversely, intense, sustained loud sound exposure results in sympathetic activation, catecholamine release, and cochlear blood flow reduction. The ensuing hypoxia is likely to damage the inner ear^25^, and therefore the sympathetic response is evolutionarily disadvantageous. In other words, the metabolic regulation of our hearing is evolutionarily unprepared for the noisy environment of industrialized society, because flight or fight responses and cochlear blood flow regulation evolved independently of loud sound, only to be linked today.

## Conclusion

Since vasodilation is induced by less intense sound stimuli^6^ than used in this study, sympathetically induced vasoconstriction apparently occurs above a certain sound threshold. The two control mechanisms, which are based in different vascular areas -lateral wall and arteriolar- possibly dominate at lower and higher SPLs respectively. This theory fits with the finding that both partial oxygen pressure^49^, and cochlear blood flow measured by LDF both acutely^16^ and chronically^50^ show such a relationship to sound intensity, and may partially contribute toward setting the sound level threshold above which the risk of hearing loss is significant.

Local feedback loops governing regulation of cochlear blood flow do not depend upon sound evoked MET channel activity. This is a most significant finding, and indicates that other candidates responsible for sound induced local cochlear blood flow regulation should be investigated, providing fresh potential in the search for therapies to treat or prevent noise induced hearing loss.

## Methods

### Ethical approval

All experiments were undertaken in accordance with protocols approved by the Institutional Animal Care and Use Committee at the Oregon Health and Sciences University. Animals were treated in accordance with the Animal Welfare Act and DHHS “Guide for the Care and Use of Laboratory Animals” and NIH guidelines.

### Animals

Male CBA/CaJ snap tested mice (RRID:IMSR_JAX:000654) were purchased from Jackson Laboratory. *Salsa* mice on the C57BL/6 background were a kind gift from Dr. Müller^12^. *Salsa* mice were crossed onto a CBA/CaJ background for 10 generations. The mice were housed under a 12 h light/dark cycle with free access to food and water and used at 7–8 weeks of age. Mice weighed 20–27 g. 78 mice were used in total. 35 mice were used for OCTA experiments. One animal was excluded due to poor imaging quality. 7 were used for CAP, CM and EP experiments. 8 were used for hair cell counts. 28 were screened using DPOAE and ABR, the data for which contributed to other studies. Following OCTA imaging or electrophysiology experiments, animals were euthanized with an overdose of anesthesia. Investigators were not blinded to the genotype of the animals.

### Genotyping protocol

*Salsa* mice harboring the homozygous *Cdh23 salsa* missense mutation were identified by polymerase chain reaction followed by restriction digest. The PCR primers (5′- CCTAAGCCAGAGGTGTTTGTG 3′ and 3′- TGTCTCACGCTGGTTCAGGAC 5’) span the point mutation in exon 22 of the Cdh23 gene. The PCR cycling parameters were: denaturation at 94 °C for 10 min followed by 72 °C for 3 min and 34 cycles of 94 °C for 30 s, 55 °C for 30 s, and 72 °C for 30 s. The resulting PCR products (765 bp) were purified on a QIAquick PCR purification column according to manufacturer’s instructions and then digested with BSAI (RRID:SCR_013517) for 4 h at 37 °C. The wild-type DNA sequence contains a BSAI recognition sequence and yields 2 restriction fragments of 465 and 300 bp in length. The *salsa Cdh23* point mutation eliminates the BSAI restriction sequence.

### Auditory brainstem response (ABR) and Distortion product otoacoustic emission (DPOAE) to assess hearing loss

ABR thresholds were measured at 16, 24, and 32 kHz in a sound attenuating chamber using the Cochlear Function Test Suite auditory evoked potential diagnostic system from Eaton Peabody Labs, and all sound stimuli were generated using a 24 bit PXI-4461 data acquisition card (DAQ) sampling at 100 kHz. To confirm that *salsa* mice on the CBA/CaJ background are deaf at p56, their ABRs and DPOAEs were recorded. Briefly, mice were anesthetized (Ketamine 30 mg/kg, Xylazine 5 mg/kg) and placed on a temperature controlled heating pad to maintain body temperature at 37 °C. Three subdermal electrodes, placed at the vertex, behind the ear, and on the leg, and a closed sound system was used to deliver 5 ms tone-pips, including 0.5 ms rise and fall times, averaged 350 times. Responses were sampled using a National Instruments PXI-6221 multifunction DAQ. Stimulus intensity was initially increased in 5 dB steps until the response began to appear; ABR threshold was defined as the lowest intensity at which an ABR Wave I response was visually identified, using the Eaton Peabody Labs ABR Peak Analysis program.

DPOAEs were recorded in the same session as ABR measurements with the same EPL system. Sound stimuli (2 tones, presented continuously by looping a finite buffer with no time gap, until a 6 dB SNR requirement is met, or the maximum number of averages is reached, f2/f1 = 1.2, L1 = L2 + 10 dB, f2 = 16–32 kHz), were delivered to the ear canal via a coupler attached to a closed acoustic system. The same individual performed all of the ABR threshold determination as well as DPOAE measurements. Following ABR and DPOAE data collection, animals were allowed to recover on a heating pad and were returned to the vivarium.

### Cochleograms to assess HC loss

Mice were deeply anesthetized and decapitated. Cochleae were immediately perfused with 4% PFA and incubated overnight at 4 °C. Following decalcification with 10% EDTA for 24 h, the cochleae were dissected and the whole mount organ of Corti segments immunolabeled with rabbit anti-myosin-VIIa (1:200, RRID:AB_10015251) overnight at 4 °C. This was followed with donkey anti-rabbit Alexa Fluor 568 secondary antibody (1:200, RRID:AB_2534017), Hoechst 33258 (RRID:AB_2651133), and Alexa Fluor 488 phalloidin (RRID:AB_2532155). Images of the immunolabeled segments were acquired on an Olympus IX81 inverted microscope fitted with an Olympus Fluoview FV1000 confocal laser microscope system. Cochlear frequency maps were generated using ImageJ (RRID:SCR_003070) to localize IHCs and OHCs based on frequency-specific regions using the Measure_Line custom plug-in (Eaton Peabody Labs, MA). The number of myosin VIIa and Hoechst positive IHCs and OHCs in each 10% length of sensory epithelium, was assessed throughout the entire length of the organ of Corti, and were plotted as a function of frequency.

### Surgical approach

Male wild-type CBA/CaJ controls or *salsa* male mice aged 7–8 weeks were anesthetized with ketamine (40 mg/kg) and xylazine (10 mg/kg). Additional doses of anesthesia were given as required to maintain an areflexive state. Subcutaneous lidocaine was also used around the scalp, throat and pinna area. The skull was exposed and the head was fixed to a head holder on a heating blanket maintained at 38 °C with a thermometer probe. The cochlea was exposed ventrally as described in^51^ with a few modifications. Briefly, the animal was tracheostomised, the pinna of the test ear resected, and the bulla exposed and quietly opened using a modified #15 scalpel, taking care not to damage the tympanic membrane. The bifurcation of the external carotid artery was routinely tied and sectioned. In a subset of animals, the SCG was identified, and the anterior outputs were sectioned with microscissors, avoiding mechanical stimulation of the vagus nerve. The head holder was angled to allow a clear path to the middle turn of the cochlea. A metal rod mounted on a manipulator was placed in position to push down upon the bulla, to minimize breathing movement artefacts.

### Electrophysiology

Compound Action Potential (CAP) and Cochlear Microphonic data were collected as in^52^. Briefly, 10 µV negative voltage deflection criterion CAP thresholds were recorded for 10 ms cosine-squared shaped (1 ms rise/fall time) tone pip stimuli (8–55 kHz) generated by a Tucker Davis System II and a DA3–4 with a sampling rate of 500 kHz. A 25 mm silver wire electrode was positioned at the edge of the round window to record CM in anesthetized mice. A silver chloride wire inserted into the neck muscle served as the reference electrode. The CM signal was digitized and multiple presentations were averaged for offline analysis.

EP was measured using a 3–5MΩ borosilicate sharp electrode, filled with 150 mM KCl. mounted on a micromanipulator, advanced through the round window and into the scala media via the organ of Corti. The electrode was connected via a headstage to an AM systems Model 3000 amplifier. The voltage was estimated from a DC coupled oscilloscope.

### Loud sound exposure protocol

Sound was delivered via an Etymotic ER•3 C loudspeaker, which formed a closed system with the ear canal via a short tube attached to a coupler. The speaker was calibrated in an artificial cavity using a Bruel and Kjaer 1/8^th^ inch 4138 microphone. The sound stimulus was low pass filtered white noise, up to 4 kHz, at 120 dB SPL for 30 minutes. The stimulus was produced by custom software written in LabVIEW 2016 64 bit (RRID:SCR_014325). This sound exposure is expected to produce acoustic trauma between 3–50 kHz^53^.

### OCTA imaging

OCTA scans were recorded using a Telesto III spectral domain optical coherence tomography system, controlled by a National Instruments 6259 DAQ. The central wavelength of the light source is 1300 nm (bandwidth 170 nm), and a 5x objective lens N.A. 0.44). The target area was the middle turn on the ventral side of the cochlea, equating to the 12–16 kHz characteristic frequency area. Live 2D structural scanning was undertaken to focus the beam, and was periodically observed to ensure no fluid was building up inside the bulla. OCTA scans were undertaken with an A-scan (2D line scan) rate of 76 kHz, 400 A-scans per B-scan (3D scan built of A-scans) over approximately 1.5 mm^2^ spanning the entire visible cochlea and part of the bulla. Each A-scan was repeated sequentially 3 times to derive the speckle variance signal. The acquisition time was 14 seconds.

Five OCTA scans were recorded in quick succession to establish a flow baseline, before the animals were subjected to the loud sound stimulus. The low pass nature of the sound exposure ensured that the majority of the cochlear partition (itself a low pass spatial filter due to the low-pass mechanical filtering properties of the entire basilar membrane with respect to the travelling wave) would respond to the noise. OCTA scans were recorded at the onset of loud sound exposure, and every 5 minutes. After 30 minutes of loud sound exposure, the measurement protocol was repeated in the absence of sound. In some experiments, the contralateral ear was measured while the ipsilateral ear was noise exposed.

### OCTA data analysis

En face images of lateral wall blood flow were produced using the SSADA algorithm^54^. Non-specific layers were segmented out using custom software, and the time-course was image registered in ImageJ using StackReg. ROI analysis was performed in MATLAB (RRID:SCR_001622), excluding non-specific signals such as those from the vibrating organ of Corti or Reissner’s membrane. Sound artefacts confirmed that the organ of Corti was responding to the stimulus. For each time point, the intensity density, which is the sum of the pixel intensity in the ROI divided by the 2D area of the ROI, was converted into a percentage change relative to the mean baseline value. Figure 1 shows an example of en face reconstruction of the flow of the lateral wall. This image was collected with no contrast agents, through the cochlear bony capsule. 5 consecutive OCTA scans were averaged in ImageJ to enhance morphology. Pixel intensity is correlated with erythrocyte velocity. Images used for analysis of flow were not averaged. Figure 3 shows examples of individual OCTA scans, with breathing noise removed, and given an arbitrary false color scale to reflect 8 bit pixel intensity, in R.

### Statistics and data analysis

Data are presented as means ± standard deviation. The overall trends of cochlear blood flow were assessed with Repeated Measures ANOVA. The within subjects variable was time. The between subjects variable was genotype (i.e. MET channel function, where the *salsa* animal, confirmed by PCR, was assumed to have none at p56 based upon characterization data), or whether the SCG had been cut. A *p* value of <0.05 was considered to be statistically significant.

## Data Availability

The datasets generated and/or analyzed during the current study are available from the corresponding author on reasonable request.
